# Emergency obstetric care in a rural district of Burundi: What are the surgical needs?

**DOI:** 10.1371/journal.pone.0170882

**Published:** 2017-02-07

**Authors:** E. De Plecker, R. Zachariah, A. M. V. Kumar, M. Trelles, S. Caluwaerts, W. van den Boogaard, J. Manirampa, K. Tayler-Smith, M. Manzi, K. Nanan-N’zeth, B. Duchenne, B. Ndelema, W. Etienne, P. Alders, R. Veerman, R. Van den Bergh

**Affiliations:** 1 Medecins sans Frontieres, Medical department, Brussels Operational Centre, Brussels, Belgium; 2 Medecins sans Frontieres, Medical department (Operational Research), Operational Centre Brussels, MSF-Luxembourg, Luxembourg; 3 International Union against Tuberculosis and Lung Disease, South-East Asia Regional Office, New Delhi India; 4 Medecins sans Frontieres, Bujumbura, Burundi; 5 Ministry of Health, Kabezi district, Burundi; 6 Medecins sans Frontieres, Operational department, Brussels Operational Centre, Brussels, Belgium; Public Library of Science, FRANCE

## Abstract

**Objectives:**

In a rural district hospital in Burundi offering Emergency Obstetric care-(EmOC), we assessed the a) characteristics of women at risk of, or with an obstetric complication and their types b) the number and type of obstetric surgical procedures and anaesthesia performed c) human resource cadres who performed surgery and anaesthesia and d) hospital exit outcomes.

**Methods:**

A retrospective analysis of EmOC data (2011 and 2012).

**Results:**

A total of 6084 women were referred for EmOC of whom 2534(42%) underwent a major surgical procedure while 1345(22%) required a minor procedure (36% women did not require any surgical procedure). All cases with uterine rupture(73) and extra-uterine pregnancy(10) and the majority with pre-uterine rupture and foetal distress required major surgery. The two most prevalent conditions requiring a minor surgical procedure were abortions (61%) and normal delivery (34%).

A total of 2544 major procedures were performed on 2534 admitted individuals. Of these, 1650(65%) required spinal and 578(23%) required general anaesthesia; 2341(92%) procedures were performed by ‘general practitioners with surgical skills’ and in 2451(96%) cases, anaesthesia was provided by nurses. Of 2534 hospital admissions related to major procedures, 2467(97%) were discharged, 21(0.8%) were referred to tertiary care and 2(0.1%) died.

**Conclusion:**

Overall, the obstetric surgical volume in rural Burundi is high with nearly six out of ten referrals requiring surgical intervention. Nonetheless, good quality care could be achieved by trained, non-specialist staff. The post-2015 development agenda needs to take this into consideration if it is to make progress towards reducing maternal mortality in Africa.

## Introduction

The Maternal Mortality Ratio (MMR) is an important measure of the status of maternal health within a given population and is defined as the number of maternal deaths per 100,000 live births over a specified time period [1]. This is an important measure as improving maternal health was one of eight Millennium Development Goals (MDGs) adopted by the international community in 2000. The fifth MDG aimed to reduce the MMR by 75% from the 1990 level by 2015 [2]. Although the number of maternal deaths dropped by 43% since 1990, it remains far from the desired target. Efforts to reduce maternal mortality thus need to be continued. In September 2015, more than 150 world leaders gathered to embrace 17 new and ambitious Sustainable Development Goals targets (SDG). The SDG 3 includes reduction of global maternal mortality ratio to less than 70 per 100,000 live births by 2030 [3].

Burundi has one of the highest MMRs in the world estimated at 740 deaths per 100,000 live births [4]. Studies by Tayler-Smith *et al* from rural Burundi showed that provision of good quality emergency obstetric care (EmOC) combined with an effective communication network and a functional ambulance transfer system for women at risk of, or with obstetric complications was associated with a substantial and rapid reduction in the MMR [5,6]. These services were supported by Médecins Sans Frontieres (MSF- an international non-governmental organization) and showed a way forward to accelerate the pace towards achieving the MDG 5 in sub-Saharan Africa where progress had been far too slow [2].

What was not assessed was the number and proportion of women accessing the EmOC facility who required surgical interventions and the types of such interventions [5,6]. Although Caesarean sections are an important part of the surgical volume of district hospitals, other life-saving emergency obstetric interventions are also performed such as laparotomy, hysterectomy, perineal and cervical tear repairs and craniotomy. Access to such surgical interventions is vital to avoiding maternal deaths and achieving set maternal health targets.

A PubMed search revealed no publications from Africa focused on women accessing EmOC who required emergency surgical interventions, the conditions that merited such interventions and the human resource cadres that performed surgery and anaesthesia. Information on the overall surgical volume at district-level settings is essential to gauge the required surgical needs and capacity. This would also inform and guide human resources (qualified obstetricians and gynaecologists and other staffs), infrastructure and training requirements.

In a rural district hospital in Burundi that offers EmOC, we thus assessed the a) characteristics of women at risk of, or with an obstetric complication and their types b) type of surgical procedure and anaesthesia performed c) human resource cadres who performed surgery and anaesthesia and d) patient outcomes at hospital exit.

## Methods

### Study design

A retrospective analysis of routine program data.

### Study setting

The study was conducted in Kabezi district, a rural district in Bujumbura, Burundi. Kabezi district has a population of about 198,000 and 9900 expected deliveries a year [7].

The district has nine health centres with a functioning maternity unit and one dedicated public district EmOC facility fully funded by MSF. The nine health centres are publically funded facilities that also received MSF support. The EmOC facility, named CURGO (Centre d’Urgence Gyneco-Obstétrique), is the only functional Emergency Obstetrical and Neonatal care (EmONC) centre in Kabezi. There is one other general hospital but without a functional operating theatre. CURGO is dedicated to obstetric emergency services.

The activities of CURGO, the ambulance transfer services and the system set-up among the nine health centres and CURGO have been described before [5,6]. The average time taken by patients for travel by ambulance to receive care at CURGO was 78 minutes (range 52–130 minutes) [5]. In brief, CURGO receives referred women, according to established criteria which includes women at risk of, or with obstetric complications **(Box 1)**. On admission to CURGO, a comprehensive package of care according to WHO standardised guidelines is offered, including Comprehensive Emergency Obstetric and Neonatal Care (CEmONC) **(Box 2)**. The CURGO centre is operational 24 hours a day, seven days a week, and all services are offered free-of-charge.

**Box 1. Referral criteria of women at risk of, or with an obstetrical complication to emergency obstetric care facility (CURGO), Burundi, 2011–2012**

**Risk factors for a complicated delivery**

■ Previous surgical intervention e.g. caesarean section
■ Previous deliveries > 5
■ First pregnancy and aged > 35 years
■ First pregnancy and women’s height < 1.5 m
■ Excessively high uterus
■ History of difficult delivery
■ History of obstetric fistula
■ General medical pathologies: severe anaemia, malnutrition, asthma, diabetes, cardiovascular or renal pathologies, infections (fever > 38°C for at least 24 hours), severe malaria

**Obstetric complications**

■ Bleeding during pregnancy
■ Post-partum haemorrhage
■ Pre-eclampsia/ eclampsia
■ Umbilical cord prolapse
■ Foetal mal-presentation
■ Prolonged labour (> 12 hours)
■ Premature rupture of membranes (with no contractions for at least 12 hours)
■ Complications of abortion (spontaneous or induced)
■ Prematurity < 37 weeks gestation
■ Intra-uterine death and uterine contractions lasting > 48 hours
■ Postpartum sepsis
■ Ectopic pregnancy

CURGO—Centre d’Urgence Gyneco-Obstétrique

**Box 2: Standard package of Comprehensive Emergency Obstetric and Neonatal Care (CEmONC) offered at the emergency obstetric care facility (CURGO), Burundi, 2011–2012**

- Antibiotics, oxytocin and anticonvulsants (magnesium sulphate)
- Manual removal of the placenta
- Misoprostol and Bakri balloon for treating
- Post-partum haemorrhage
- Removal of retained products following abortion
- Instrumental vaginal delivery
- Surgery (caesarean section, hysterectomy, laparotomy)
- Safe blood transfusion
- Newborn care including care for sick and low birth weight newborns (essential medicines, blood transfusion)
- Oxygen, basic and advanced resuscitation

CURGO—Centre d’Urgence Gyneco-Obstétrique

### Patient flow, surgery and surgical infrastructure

The activities are assured by the presence of national general practitioners who are the first-line and whose competencies in emergency obstetric surgery are enhanced through on-site training by expatriate gynaeco-obstetricians. Expatriate gynaeco-obstetricians also serve as second-line support for cases that cannot be handled by the general practitioners. Women transferred to CURGO are first assessed by a trained midwife and are reviewed by a general practitioner with surgical skills. An obstetrician reviews the case if there is a need for more advanced review. If a woman requires surgery a written consent form is signed, the operating room (OR) staff are informed and the woman is prepared for surgery by midwives. This involves hygiene, urinary catheter insertion and insertion of an intra-venous line. The woman is then prepared by an anaesthesia nurse or anaesthetist in the operating theatre. Once the woman is deemed ready for surgery, this is performed by a general practitioner with surgical skills and may involve an obstetrician depending on the level of complexity. Support is provided by an instrument nurse and midwife. After surgery, the patient is transferred to a post-operative room and when stable to the obstetrics ward. The human resources allocated to surgical activities are shown in Table 1.

**Table 1:** The allocated human resources required to offer surgical activities at an emergency obstetric care facility (CURGO), Burundi, 2011–2012.

Human resources (HR) Cadre	Total HR in CURGO	No HR allocated to surgery/day/ shift
General practitioner (GP)	4	1
Obstetrician	1	1
Anaesthetist	1	1
Instrument nurse	5	1
Nurse anaesthetist	4	1
Head nurse	1	1
Midwife	12	1
Cleaners	3	1

CURGO—Centre d’Urgence Gyneco-Obstétrique; HR–Human resources.

For anaesthesia management it was decided to work only with nurse-anaesthetists, with the possibility to refer for advanced anaesthesia and post-operative intensive care to hospitals in the capital city. The competences of the local nurse-anaesthetists were supported and evaluated throughout a tailored anaesthesia training conducted by a senior anaesthesiologist. All through, the competences of the nurse-anaesthetists were monitored by an anaesthesia report book and yearly supervision.

**Fig 1:**
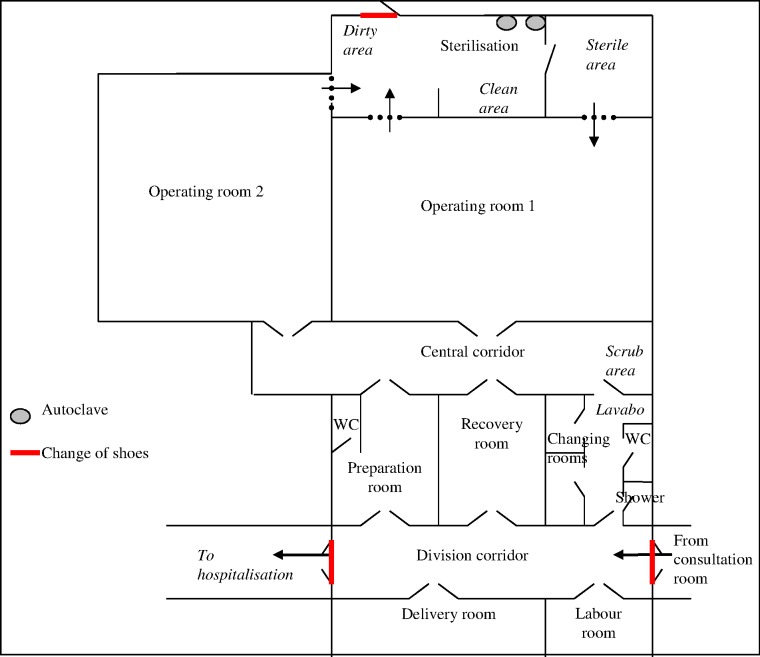
General layout of the sterilisation service and the Operating Department, CURGO, Kabezi, Burundi.

The CURGO centre is a building that was newly constructed by MSF. The OR’s are located inside the Operation Department (OD) and in order to facilitate referral, they share a common corridor with the delivery and labour unit. The OD has 2 OR’s, each equipped with one operating bed and supporting equipment. There is a preparation room where the pre-operative anaesthesia visit is held and a joint corridor with the surgical washing point. There are also toilets and a shower. Finally, there is a recovery room with a two bed capacity, and two locker rooms. The operation room is connected with the sterilisation area by two windows, one to give the contaminated material and other to receive the sterilised material. Surgical instruments, consumables, oxygen cylinders and concentrators are available as well as suction equipment—all of which are provided by MSF. Resuscitation equipment for the mother and newborn is also available. There is a 24 hour stand-by generator and independent water supply tank. The general layout of the operations department is shown in Fig 1.

### Study population and period

The study included all women who arrived at the CURGO facility at risk of or with an obstetric complication during the years 2011 and 2012. The study was conducted between July 2013 and April 2014.

### Data and analysis

Two databases were used in this study: a dedicated and individualized database of all patients transferred for emergency obstetric care, covering admission, treatment and discharge characteristics of patients, and an individualized operating department register for collection of all peri-operative data. Data for the transfer database were cross-validated with patient files and entered by one dedicated and trained data entry clerk who was well supervised. Data for the surgical database were entered by the same date entry clerk and underwent extensive checks and corrections by the surgical referent in MSF-headquarters. The databases were cross-validated against each other.

Type of surgical procedure was categorized into major (caesarean section, hysterectomy, laparotomy, curettage and other obstetrical interventions including cervical tear suture, repair of third and fourth perineal tear and craniotomy) and minor procedures (episiotomy, first and second- degree perineal sutures, symphysiotomy and manual vacuum aspiration (MVA)). Hospital exit outcomes were standardized and included discharge, died, absconded and referred to tertiary care. Descriptive analysis was performed using EpiData Analysis software (version 2.2.2.182, EpiData Association, Odense, Denmark)

### Ethics

This study fulfilled the MSF Ethics Review Board, (Geneva, Switzerland) approved criteria for analysis of routinely collected program data and has also met the same requirements of the Ethics Advisory Group of the International Union against Tuberculosis and Lung Disease, Paris, France and has received approval. Patient records and information was anonymized and de-identified prior to analysis.

## Results

### Characteristics of women at risk of, or with an obstetric complication and their types

A total of 6084 women were transferred to CURGO for EmOC, of whom 2534 (42%) underwent a major surgical procedure while 1345 (22%) required a minor procedure **(Table 2)**. 208 women underwent both minor and major surgery. Standardizing these numbers by coverage population implies 64 major obstetric surgical procedures performed /10,000 population/year. Among all women transferred for EmOC, 2413 (40%) did not require any surgical procedure.

**Table 2:** Proportion of women referred to an emergency obstetric care facility (CURGO) and who underwent surgical procedures, stratified by age, gestational age, gravida, abortion, parity and diagnosis, Kabezi, Burundi, 2011–2012.

	Total n	Major surgical procedure^a^ n (%)	Minor surgical procedure^b^ n (%)
Total	6084	2534 (42)	1345 (22)
Age			
13–19	767	305 (40)	265 (35)
20–29	3326	1488 (45)	749 (23)
30–39	1609	612 (38)	248 (15)
40+	310	108 (35)	70 (23)
Unknown	72	21 (29)	13 (18)
Median, years (IQR)	25 (21–30)	25 (21–30)	23 (20–29)
Gestational age			
≥36	3613	1602 (44)	642 (18)
32–35	313	88 (28)	42 (13)
26–31	222	45 (20)	29 (13)
<26	854	83 (10)	422 (49)
Not recorded	1084	347 (32)	211 (19)
Gravida			
1	1702	639 (38)	563 (33)
2–6	3337	1271 (38)	607 (18)
>6	1024	253 (25)	176 (17)
Not recorded	21	2 (10)	0 (0)
Parity			
0	1766	674 (38)	592 (34)
1–6	3768	1372 (36)	664 (18)
>6	531	116 (22)	90 (17)
Not recorded	21	3 (14)	0 (0)
Abortion			
0	5170	1910 (37)	1149 (22)
1–3	829	235 (28)	185 (22)
>3	59	15 (25)	10 (17)
Not recorded	28	5 (18)	2 (7)
Uterine rupture	73	73 (100)	2 (3)
Extra-uterine pregnancy	10	10 (100)	1 (10)
Pre-uterine rupture	111	108 (97)	4 (4)
Foetal distress	183	178 (97)	4 (2)
Cord prolapse	78	73 (94)	3 (4)
Uterine scar	404	365 (90)	14 (3)
Malpresentation	273	219 (80)	19 (7)
Post-operative infection	23	17 (74)	-
Dystocia	1420	840 (59)	259 (18)
Bleeding	309	175 (57)	53 (17)
Not recorded	59	22 (37)	-
Twin pregnancy	142	49 (34)	23 (16)
Pathology during pregnancy	118	40 (35)	27 (23)
Sepsis	21	6 (29)	-
Others^C^	269	76 (28)	34 (13)
Abortion^d^	842	159 (19)	510 (61)
Preterm delivery	263	25 (10)	34 (13)
Normal delivery	1053	76 (7)	356 (34)

a. Surgical intervention: Caesarean section, hysterectomy, curettage, laparotomy, cervical tear repair, repair of 3rd to 4^th^ degree perineal tear, craniotomy; A person who had more than one major surgical procedure was counted as one for the purpose of this analysis. A patient may have had both major and minor surgical procedures.

b. Minor surgical procedure: Manual vacuum aspiration, episiotomy, repair of 1st and 2^nd^ degree perineal tear, symphysiotomy; A person who had more than one minor surgical procedure was counted as one for the purpose of this analysis. A patient may have had both major and minor surgical procedures.

c. Others: Medical conditions, fistula, premature rupture of membranes >12 hours.

d. Abortion: Threatened, complicated and non-complicated abortion CURGO—Centre d’Urgence Gyneco-Obstétrique.

All cases of uterine rupture (73) and extra-uterine pregnancy (10) required major surgery, as well as the great majority of women with pre-uterine rupture and foetal distress (97% of 111 and 178 respectively). The two most prevalent conditions requiring a minor surgical procedure were abortions (61%) and normal delivery (34%).

### Number and type of major surgical procedure and anaesthesia performed

Out of 2544 major surgical procedures, 2057 (81%) were caesarean sections (**Table 3)**. Of these, 1650 (65%) required spinal anaesthesia while 578 (23%) were general anaesthesia. The most common condition requiring general anaesthesia was hysterectomy (89%, **Table 3**).

**Table 3:** Type of major surgical procedures, stratified by the type of anaesthesia performed at an emergency obstetric care facility (CURGO), Burundi, 2011–2012.

Type of surgical procedure	Type of anaesthesia				Total
	General n (%)*	Spinal n (%)	Local n (%)	Combined n (%)	
Caesarean section	365 (18)	1568 (76)	-	124 (6)	2057
Hysterectomy immediately after Caesarean section	13 (62)	6 (29)	-	2 (9)	21
Hysterectomy	8 (89)	0 (0)	-	1 (11)	9
Laparotomy (ectopic pregnancy)	9 (64)	4 (29)	-	1 (7)	14
Curettage	135 (62)	28 (13)	50 (23)	6 (3)	219
Other obstetrical interventions (Cervical tear suture, Repair of perineal tear^a^, craniotomy)	49(22)	45 (20)	124 (55)	3 (1)	224 ^b^
**Total**	578 (23)	1650 (65)	173 (7)	123 (5)	2544

a. 3rd to 4th degree.

b. Type of anesthesia was not recorded for three interventions.

* These indicate row percentages

CURGO—Centre d’Urgence Gyneco-Obstétrique.

**Fig 2:**
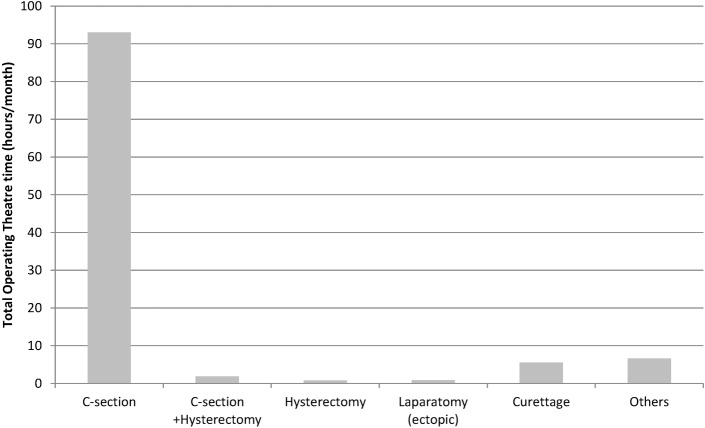
Total operating theatre time by major surgical procedure performed at an emergency obstetric care facility (CURGO), Burundi, 2011–2012.

**Fig 3:**
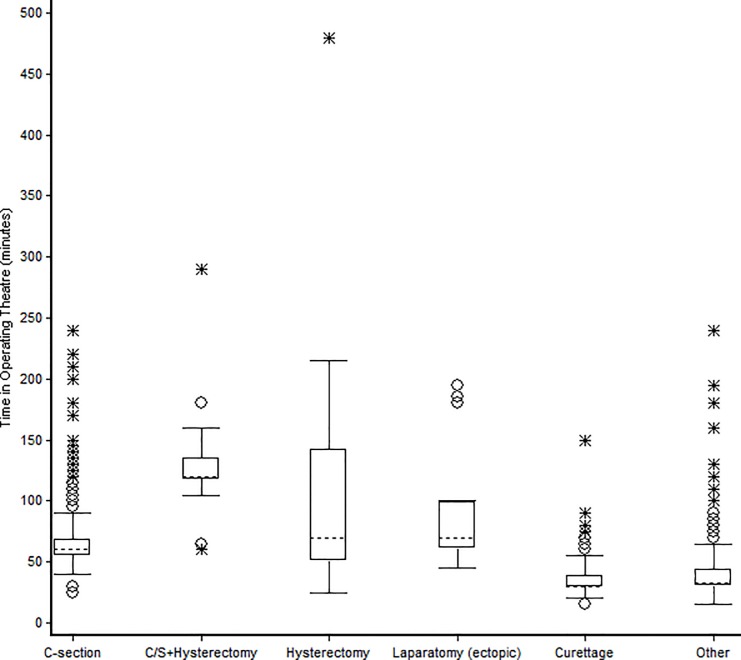
Median operating theatre time of major surgical procedures performed at emergency obstetric care facility (CURGO), Burundi, 2011–2012.

**Fig 2**shows the cumulative OR occupancy time (hours per month) by type of major surgical procedure. The total OR occupancy time per month was 109 hours (mean of four hours of OT time/day). **Fig 3**shows the median OR time utilized for one intervention by type of major surgical intervention. The longest OR time was for hysterectomies and laparotomies.

### Human resource cadres who performed surgery and anaesthesia

**Table 4**shows the human resource cadres performing major surgery and anaesthesia. Out of 2544 interventions 2341 (92%) were performed by general practitioners with surgical skills while 2451 (96%) anaesthesias were conducted by anaesthesia nurses.

**Table 4:** Proportion of major surgical procedures, stratified by human resource cadres, performed at an emergency obstetric care facility (CURGO), Burundi, 2011–2012.

	Surgery performed by			Anaesthesia performed by		
Type of surgical procedure	Obstetrician (%)*	General practitioner with surgical skills n (%)	Total	Anaesthetist n (%)	Anaesthesia nurse n (%)	Total
Caesarean section	163 (8)	1894 (92)	2057	73 (3)	1984 (96)	2057
Hysterectomy immediately after caesarean section	8 (38)	13 (62)	21	1 (5)	20 (95)	21
Hysterectomy	2 (22)	7 (78)	9	-	9 (100)	9
Laparotomy (ectopic pregnancy)	6 (43)	8 (57)	14	2 (14)	12 (85)	14
Curettage	3 (1)	216 (99)	219	10 (5)	209 (95)	219
Other obstetrical interventions (Cervical tear suture, repair of perineal tear^a^, craniotomy)	21 (9)	203 (91)	224	7 (3)	217 (97)	224
**Total**	203 (8)	2341(92)	2544	93 (4)	2451 (96)	2544

a. 3rd to 4th degree.

* These indicate row percentages; CURGO—Centre d’Urgence Gyneco-Obstétrique.

### Patient operative and hospital outcomes

Post-operative infection was recorded in 87 (4%) of all interventions. **Table 5**shows patient outcomes at hospital exit stratified by type of surgical procedure. Of 2534 admissions for major surgical procedures, 2467 (97%) were discharged (uneventfully) and there were 2 (0.1%) registered deaths.

**Table 5:** Patient outcomes at hospital exit at an emergency obstetric care facility (CURGO), Burundi, 2011–2012.

	Total admission	Discharged n (%)	Referred to tertiary care n (%)	Died n (%)	Absconded^a^ n (%)	Not recorded n (%)
Major surgical procedure	2534	2467 (97.4)	21 (0.8)	2 (0.1	6 (0.2)	38 (1.5)
Minor surgical procedure	1137	1135 (99.8)	2 (0.2)	-	-	-
No surgery done	2075	1890 (78.3)	99 (4.1)	4 (0.2)	72 (3)	10 (0.4)
**Total**^b^	5746	5492 (95.5)	122 (2.1)	6 (0.1)	78 (1.3)	48 (0.8)

a. Patient left the emergency obstetric care facility without medical consent.

b. 338 patients (14%) were not referred to CURGO and no surgical procedure was performed.

CURGO—Centre d’Urgence Gyneco-Obstétrique.

## Discussion

This is one of the first studies from a rural district hospital setting in Africa that has quantified the type of emergency obstetric conditions for which surgical interventions were required. About four in ten women transferred for EmOC required major surgery which was performed by trained general practitioners and nurse anaesthetists (non-specialists) and hospital exit outcomes were very good with only two reported maternal deaths.

This finding is particularly encouraging since the facility only received obstetric referrals who were at risk of, or already manifesting an obstetric complication—these patients were thus at high risk of maternal death.

The strengths of the study are that obstetric complications were carefully identified, dedicated databases existed both for obstetric care and for surgery and these were closely supervised. Furthermore, the findings come from a rural district hospital facility in a country with a high MMR and thus have wider public health implications.

The study limitations are that we do not know if any deaths occurred after hospital discharge and post-operative infection was recorded only until hospital exit. This hospital was also better resourced than most other hospitals in similar settings since it benefited from support of MSF and this is a consideration for replicability [6].

The findings of this study have a number of policy and practice implications. First, the MDGs and SDGs do not explicitly mention surgical care as a component of global health care and maternal health [8]. Our study reveals that six in ten referred women needed some sort of obstetric surgical intervention. Catering for this need is thus essential to reduce maternal mortality and achieve the set maternal health targets. The post 2015 development agenda needs to take this consideration on board.

Second, two previous studies from the same setting showed a rapid reduction in MMR associated with good quality obstetric emergency care and an efficient patient ambulance transfer system [5,6]. This study now shows that good quality care can be provided largely by non-specialist staff who are well trained and supervised. Similar evidence of non-specialists providing comparable surgical care to those by obstetricians has been reported from Mozambique and Malawi [9,10]. Such task-sharing is the way forward in ensuring access to surgical services in other sub-Saharan Africa countries struggling to reduce maternal mortality and faced with dire shortages of specialized obstetricians and anaesthetists [11]. The experience may also be useful to bridge the overall global gap, with an estimated two billion people, (i.e. 30% of the global population) lacking access to surgical services and millions being subjected to unsafe anaesthesia procedures [12,13].

Third, the emergency obstetric facility performed 64 major obstetrical procedures per 10,000 population per year. The respective rates in Tanzania, Uganda and Mozambique were much lower at six, 14 and 15 [13]. Obstetric emergency surgical coverage was thus four to ten times higher in our setting. This is tribute to a well-functioning centralized EmOC facility linked to an effective referral and patient transfer system [5].

Fourth, about one in ten major surgeries was performed for foetal indications (foetal distress and cord prolapse) and this may have had a direct impact not only on the mothers health, but importantly on reducing neonatal mortality–the latter comprising 40% of all deaths in under-fives and thus important for the MDG 4 [14].

Finally, despite the diversity of surgical procedures, we had a post-operative infection rate of about four percent. Although we aim for ‘zero’ post-operative infections, this is still an encouraging indicator of the overall quality of surgical and post-operative nursing care in this setting. This compares well with data from a multicentre study where the average post-operative infection rate was 7.3% and ranged between 1.7 and 10.4% [15].

In conclusion, we have shown that the overall surgical volume in an emergency obstetric facility in rural Burundi is high and diverse, but good quality care can be achieved by trained non-specialist staff. The post 2015 development agenda needs to take this into consideration if it is to make further pace in efforts towards reducing maternal mortality in Africa.
